# Supplementary material for: Water, sanitation, and hygiene in selected health facilities in Ethiopia: risks for healthcare acquired antimicrobial-resistant infections
Source: Front Public Health. 2024 Dec 2;12:1478906. doi: 10.3389/fpubh.2024.1478906 (PMC11647025; doi:10.3389/fpubh.2024.1478906)
Supplement: Supplementary file 1 [file Data_Sheet_1.pdf]

Water, sanitation, and hygiene in selected health facilities in Ethiopia: risks for healthcare acquired antibiotic resistant infections

Supplemental file

Table S1 List of bacteria isolated from environmental and water samples from health facilities in Bulle district

No	Site in the healthcare setting	Gram Negative	Gram positive
**Surface samples**			
1	Pediatric OPD (Bed1)	*Acinitobacter* Spp, *Stentophomonas maltophilia*	*Baccillus* spp
2	Pediatric OPD (Bed2)	*E. coli*,	
3	Pediatric OPD (table)	*E. coli*, *E. hermani*, *Acinitobacter* spp	
4	Pediatric OPD (table)	*Burkholderia* spp, *Comamonas testosteroni*, Non-fermenter	
5	Chronic OPD	*E. coli*, *Citrobacter* spp	
6	ANC	Non-fermenter,	*Baccillus* Spp
7	EPI	*Klebsiella* spp, *E. coli*	
8	Family plan	*K. pneumoniae*, *K. ozeanea*	
9	Delivery	*E. coli*, *Acinitobacter* spp	
10	Reception	*E. coli*, *K. aerogens*	
11	Emergency	*Acinitobacter* Spp	*S. aureus*
12	Procedure room	*Pseudomonas* spp	*S. aureus*, *Bacillus* species
13	Emergency Trial	*E. cloacae* , *Klebsiella* spp	*S. aureus*,
14	NICU	*Klebsiella* spp,	S. aureus
15	Delivery room		*Bacillus* spp
16	Delivery room Gaun	*E. coli*	*S. aureus*
17	Postnatal 1	*E. coli*	*Bacillus* spp
18	Postnatal 2	*E. coli*	*S. aureus*
19	Operation Room		*Bacillus* spp
20	Toilet door hand	*E. coli*, *Proteus mirablis*	
21	Stabilization 1	*Klebsiella spp*, Non Lactose fermenter	*S. aureus*
22	Stabilization 2	*E. coli.*	*S. aureus*
23	Adult female medical ward	*E. coli*, *Klebsiella spp*	
24	Pediatric medical ward	*Klebsiella spp*, *Acinitobacter* spp	*S. aureus*
25	Surgical ward	*Acinitobacter* spp	*S. aureus*
26	Laboratory	*Acinitobacter* spp, *Klebsiella spp*	*S. aureus*, *Bacillus* species
27	Sterilization		*S. aureus*, *Bacillus* spp
28	Door hand (HP)		*Bacillus* spp
29	Table (HP)		*Bacillus* spp
**Water samples**			
31	Delivery ward water	*E. coli*	
32	Tanker water		*Bacillus spp*
33	Small tanker water	*Non Lactose fermenter*, *Klebsiella spp*	
34	Medical ward water	*Klebsiella spp*, *Citrobacter spp*	
35	Rain water health post	Non Lactose fermenter,	
36	Tanker Seko health post		*Bacillus spp*
37	Borehole Seko health post		*Bacillus spp*

ART, antiretroviral therapy; EPI, expanded programme on immunization; HC, health center; ICU, intensive care unit; OPD, outpatient department

Table S2 Bacteria isolated from environmental and water samples from health facilities in Doyo Gena district

No	Site in the healthcare setting	Gram Negative	Gram positive
1	Reception	*Acinitobacter* spp	*Bacillus* spp
2	Emergency	*Acinitobacter* spp, *Alcaligenes* spp	*Bacillus* spp, *Staphylococcus* spp
3	Emergency procedure	*Enterobacter cloacae*	*Bacillus* spp, *Staphylococcus* spp
4	Under 5 Emergency	*Acinitobacter* spp	*Bacillus* spp
5	NICU	*Acinitobacter* spp	*Bacillus* spp
6	medical ward	*E. coli*, *Providencia rettigi*	*Bacillus* spp
7	surgical ward	*E. coli*, *Acinitobacter* spp	
8	female medical ward	*E. coli*, *K. oxytoca, Alcaligeues faecalis*	
9	Door medical ward	*Citrobacter* spp, *Alcaligeues faecalis*	*Staphylococcus* spp
10	Gynecology ward	*E. coli*, *K. aerogenes*	
11	male surgical ward	*E. coli*, *Pseudomonas aeruginosa*	*Staphylococcus* spp
12	pediatric ward	*E. coli*, *Pseudomonas aeruginosa*	
13	Laboratory	*E. coli*, *Pseudomonas aeruginosa*	*Baccillus* Spp
14	Card room	*E. coli*	*Baccillus* Spp
15	Pharmacy	*E. coli*	*Baccillus* Spp
16	family planning	*Pseudomonas aeruginosa*, *Alcaligenes* spp	*Baccillus* Spp
17	ANC	*Klebsiella aerogenes*	
18	Operation Room	*E. coli*, *Enterobacter cloacae*	*Staphylococcus* spp
19	Delivery	Citrobacter spp, Klebsiella spp	*Staphylococcus* spp
20	Door Zeraro HP	*K. oxytoca*, *Alcaligenes* spp	
21	Table Zeraro HP	*E. coli*, *Citrobacter* spp	
22	Door Leino HP	*E. coli*	*Baccillus* Spp
23	Table Leino HP	*Enterobacter* spp, *Alcaligenes* spp	*Baccillus* Spp
24	Delivery Sararo HC	*K. oxytoca*	*Staphylococcus* spp
25	Emergency SararoHC	*K. pneumonia*, *Citrobacter spp*	
26	Abortion care SararoHC	*K.pneumoniae*	
27	ANC and FP Sararo HC	*Alcaligenes facalis*	
28	Reception sararo HC		
29	Under 5 OPD Sararo HC	*Morganella* spp	*Baccillus* Spp
30	Adult OPD Sararo HC	*Morganella* spp	*Baccillus* Spp
**Water samples**			
31	Hospital water tanker 1	*E. coli*, *Citrobacter* spp	
32	Hospital water tanker 2	*K. pneumoniae*, *Pseudomonas* spp	
33	HC water (rain water)	*E. coli*, *Pseudomonas* spp	

ART, antiretroviral therapy; EPI, expanded programme on immunization; HC, health center; ICU, intensive care unit; OPD, outpatient department

Table S3 List of bacteria isolated from environmental and water samples from health facilities in Bidre district

No	Site in the healthcare setting	Gram Negative	Gram Positive
1	Emergency	*Acinitobacter* spp, *Pseudomonas* spp,	*Bacillus* spp
2	Observation	*Enterobacter cloacae*, *Klebsiella* spp	*Bacillus* spp
3	Procedure	*E. coli*, *Acinitobacter* spp,	
4	Wards Door	*Acinitobacter* spp	
5	Gynecology	*Citrobacter* spp,	*Staphylococcus* spp
6	Will chair	*E. coli*	
7	Pre-term	*Edwardians* spp	
8	Obstetric room	*Klebsiella* spp, *Acinitobacter* spp,	*Bacillus* spp *Enterococcus* spp *Staphylococcus* spp
9	Delivery	*Klebsiella* spp, *Acinitobacter* spp,	*Bacillus* spp
10	Post-natal	*E. coli*	*Enterococcus* spp, *Staphylococcus* spp
11	Male surgical	*E. coli* , *Klebsiella* spp, Non- lactose fermenter	*Staphylococcus* spp
12	Isolation (pediatric)	Non- lactose fermenter, *Enterobacter* spp	*Bacillus* spp, *Staphylococcus* spp
13	Pediatric ward	*Klebsiella* spp, Non- lactose fermenter, *Enterobacter* spp	
14	stabilization phase I	*Klebsiella ozene*, 2Non- lactose fermenter	*Bacillus* spp *Staphylococcus* spp
15	toilet door	*E. coli*	
16	Pediatric OPD	2 Non- lactose fermenter	*Bacillus* spp *Staphylococcus* spp
17	Adult OPD	Non- lactose fermenter, *Klebsiella* spp	*Staphylococcus* spp
18	Reception	Non- lactose fermenter	*Staphylococcus* spp
19	Laboratory	*E. coli*	*Staphylococcus* spp
20	Female surgical ward	*Klebsiella* spp, *Enterobacter* spp,	*Staphylococcus* spp
21	Wards Door hands	*Klebsiella* spp,	*Staphylococcus* spp
22	Psychiatric room	*Enterobacter* spp	*Staphylococcus* spp
23	Female medical ward	*Enterobacter* spp	*Staphylococcus* spp
24	Staff cafeteria	*Enterobacter* spp	*Staphylococcus* spp
25	Pharmacy	Non- lactose fermenter	
26	Autoclave		
27	HC Adult OPD	Non- lactose fermenter	*Staphylococcus* spp
28	HC Emergency OPD	*E. coli*, Non- lactose fermenter	
29	HC laboratory	*Acinitobacter* spp	*Staphylococcus* spp
30	HC ART clinic	*E. coli*, Non- lactose fermenter	
31	HC EPI	Non- lactose fermenter	*Staphylococcus* spp
	**Water samples**		
32	Bidre hospital Borehole (Water)	Non- lactose fermenter,	
33	Bidre health center water from tanker	Non- lactose fermenter, *Citrobacter* spp	

ART, antiretroviral therapy; EPI, expanded programme on immunization; HC, health center; ICU, intensive care unit; OPD, outpatient department

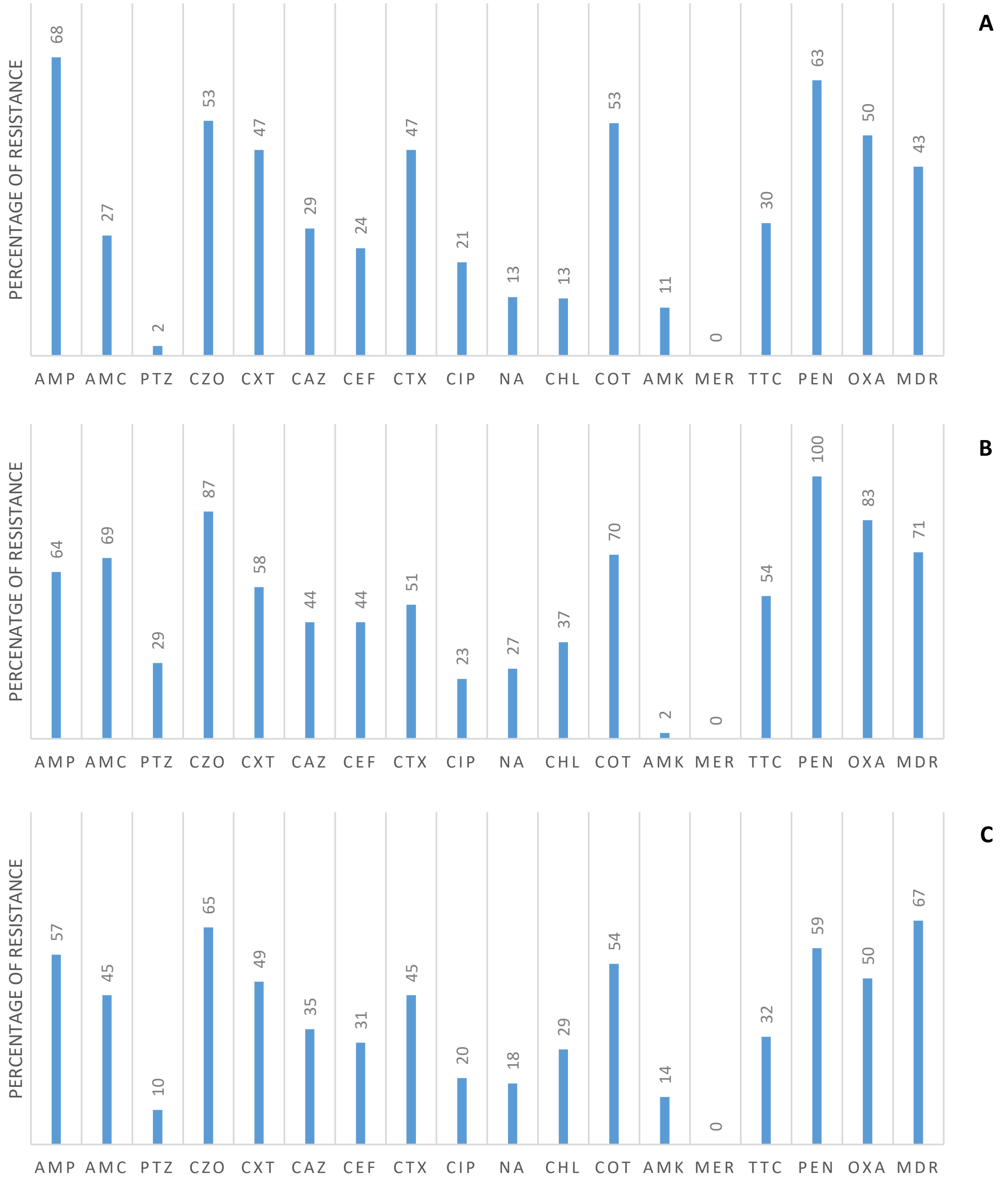

Figure S1 Antibiotic susceptibility profile and multidrug resistance patterns in DoyoGena (A), Bulle (B), and Bidre (C)

Percentage of resistance of 70 bacterial isolates identified from healthcare surface environmental and water samples of Bulle district of Gedo zone SPNN region of Ethiopia according to the CLSI disk diffusion breakpoints. Resistance was defined as isolates with intermediate resistance and complete resistance inhibition zone size. Antibiotics tested were ampicillin (AMP), amoxicillin-clavulanate (AMC), pepracillin with tazobactum (PTZ), cefazolin (CZO), cefuroxime (CXT), ceftazidime (CAZ), cefepime (CEF), cefotaxime (CTX), ciprofloxacin (CIP),naldixix acid (NA, chloramphenicol (CHL), Cotrimoxaxol (COT) Amikacin (AMK), Meropenem (MER),tetracycline (TTC), Penicillin (PEN) and oxacillin (OXA). MDR is to indicate the rate of Multidrug resistant bacterial isolates
